# Supplementary material for: A fusion protein comprising pneumococcal surface protein A and a pneumolysin derivate confers protection in a murine model of pneumococcal pneumonia
Source: PLoS One. 2022 Dec 7;17(12):e0277304. doi: 10.1371/journal.pone.0277304 (PMC9728834; doi:10.1371/journal.pone.0277304)
Supplement: S1 Appendix — The cytokine production was analyzed in the BALF after 6, 12, 24 and 168 hours after infection using the Cytometric Bead Array (CBA, BD Biosciences). (DOCX) [file pone.0277304.s001.docx]

**S1 Appendix – Cytokine production analysis by CBA kit.** The cytokine production was analyzed in the BALF after 6, 12, 24 and 168 hours after infection using the Cytometric Bead Array (CBA, BD Biosciences).
